# Feasibility of a home-based physiotherapy intervention to promote post-stroke mobility: A randomized controlled pilot study

**DOI:** 10.1371/journal.pone.0256455

**Published:** 2022-03-07

**Authors:** Ameerani Jarbandhan, Jerry Toelsie, DirkJan Veeger, Robbert Bipat, Luc Vanhees, Roselien Buys

**Affiliations:** 1 Dept. of Anatomy, Anton de Kom University of Suriname, Paramaribo, Suriname; 2 Dept. of Physiology, Anton de Kom University of Suriname, Paramaribo, Suriname; 3 Dept. of Biomechanical Engineering, Delft University of Technology, Delft, the Netherlands; 4 Dept. of Rehabilitation Sciences, KU Leuven, Leuven, Belgium; Prince Sattam Bin Abdulaziz University, College of Applied Medical Sciences, SAUDI ARABIA

## Abstract

**Objectives:**

Home-based physiotherapy interventions to improve post-stroke mobility are successful in high-income countries. These programs require less resources compared to center-based programs. However, feasibility of such an intervention in a low and middle-income setting remains unknown. Therefore, the SunRISe (Stroke Rehabilitation In Suriname) study aimed to assess feasibility and preliminary effectiveness of a home-based semi-supervised physiotherapy intervention to promote post-stroke mobility in a low resource setting.

**Design:**

Prospective randomized controlled trial.

**Methods:**

Chronic stroke patients were recruited and randomized into either an intervention group (IG (N = 20)) or a control group (CG (N = 10)). The IG received a 3-days-a-week home-based physiotherapy program that was supervised in the first 4 weeks and tele-supervised during the second 4 weeks. The physiotherapy program consisted of walking as well as functional and mobilization exercises. The CG received usual care. Feasibility outcome measures included adherence, patient satisfaction and safety. Efficacy measures included functional exercise tolerance (six-minute walking test (6MWT), functional balance (Berg Balance Score (BBS), upper extremity (UE) function (Disabilities of the Arm, Shoulder and Hand (DASH) Questionnaire), and UE strength ((non-)paretic handgrip (HG) strength). Two-way analysis of variance was used for data analysis.

**Results:**

Thirty participants (61.8 ± 9.2 years old, 13 men) were enrolled in the study. The intervention was completed by 14 participants (70%). Adherence was affected by rainy season associated infrastructural problems (n = 2), the medical status of participants (n = 3) and insufficient motivation to continue the program without direct supervision (n = 1). No adverse events were noted and participants were satisfied with the program. Functional exercise tolerance (57.2 ± 67.3m, p = 0.02) and UE function (-9.8 ± 15.2, p = 0.04) improved in the IG compared to no change in the CG. HG strength was unaltered and a ceiling effect occurred for BBS.

**Conclusion:**

Our home-based semi-supervised physiotherapy intervention seems safe, associated with moderate to high levels of engagement and patient satisfaction and results in functional improvements.

## Introduction

Stroke rehabilitation reduces the risk for recurrent stroke, improves mobility, independency during Activities of Daily Life (ADL) as well as quality of life [1–7]. Hence, various types of physiotherapeutic programs for rehabilitation have been assessed for their success rate [7–12].

During home-based physiotherapy interventions the physiotherapist (PT) usually visits a patient at home to conduct the intervention [13, 14], as opposed to tele-physiotherapy where the objective is to allow medical experts to manage their patients through telecommunication technologies without requiring the patient to leave their home, or the PT to be onsite [15]. Several studies [6, 7, 12, 14, 16, 17] have shown that home-based therapy, including tele-physiotherapy, improved independence compared to conventional care and is more cost-effective compared to physiotherapy in the hospital [12, 18]. Today, home-based and tele-physiotherapy programs are of increasing interest and appear to have a high success rate for improving post-stroke mobility [6, 7, 12–14, 16, 17]. However, their applicability in LMIC has yet to be documented [6] since most of these home-based physiotherapy studies have been conducted in High Income Countries (HIC) [6, 7, 12, 16, 19–22]. Moreover, due to high costs and limited availability of traditional rehabilitation services, stroke rehabilitation is underutilized in LMICs. Poor mobility due to bad road conditions, traffic jams and poverty further limit the accessibility of rehabilitation services. Only few studies have looked in to this in a LMIC setting; the ATTEND home-based rehabilitation trial (India) [23], the SMART tele-rehabilitation trial (India and Nigeria), [24] the RECOVER trial (China) [25] and feasibility study F@ce (Uganda) [26]. The latter comprised of an 8-week mobile phone supported, family-centered intervention that was found to improve ADL and self-efficacy of ADL. The ATTEND trial [23] inquired for dependency and mortality of the patient at 6 months and found no difference between the control and the intervention group when care was shifted to family at home in comparison to usual care. Through the RECOVER mobile phone app, acute stroke survivors would be followed for improvement in ADL via a 2-year randomized controlled trial. The SMART trial comprised of an eight-week DVD-based intervention for improvement of functional outcomes [24]. Results for the RECOVER and SMART trials have not yet been published. Today, no structural home-based or tele-physiotherapy program is available for chronic stroke survivors in Suriname. Currently, reimbursed therapy sessions at the rehabilitation center in the capital of Suriname are offered for a maximum of ten sessions in one year. Suriname is contextually unique: socio-economic barriers, cultural barriers, uncoordinated care, under-diagnosis, low awareness, lack of research, etc. limit the capacity to develop successful strategies against the rising burden of disease [27]. From this point of view, the SunRISe (Stroke Rehabilitation In Suriname) project was initiated, in which we developed an 8-week home-based semi-supervised physiotherapy program to improve functioning of chronic stroke survivors in the urban area of Suriname. The aim of this pilot study was to assess the feasibility and safety of this program. Additionally, the clinical efficacy of this approach was explored. We hypothesize that this program will be feasible in a low-income setting and will induce a clinically important improvement. Furthermore, this study will generate efficacy estimates to inform sample size calculation and best primary outcome measures for designing a properly powered randomized controlled trial in the future.

## Methods

### Participants

A convenience sample of 30 chronic stroke patients was randomly recruited from the database of the Academic Hospital Paramaribo (AZP) and the community between April 2016 and April 2017. Potential eligible patients with stroke, and some of their general physicians, were called in order to evaluate eligibility for the baseline exercise test according to the inclusion and exclusion criteria. Inclusion criteria were chronic stroke patients who had a stroke at least 6 months ago, had medical clearance to participate in a moderately intense exercise program, were living at home, were not already undertaking regular exercise or physiotherapy, had a mild to moderate stroke deficit (defined by Fugl Meyer test score of 27 to 90 for upper and lower extremities [4]), had a Functional Ambulation Classification score ≥3 and were able to understand measurement procedures (defined as Mini Mental State Examination score >24 [5]). Exclusion criteria were: absolute and relative contraindications to exercise testing (according to the American Heart Association guidelines) [28] as well as other neurological deficits leading to disability. Contractures, hypertony or spasticity were no reason for exclusion as long as the participants adhered to all above mentioned inclusion and exclusion criteria. The Board of Ethics and Research of Ministry of Health in Suriname approved this study (VG 023–15) and all participants gave their written informed consent. The study was prospectively registered on ClinicalTrials.gov with identifier: NCT02717715.

### Study design and outcome assessment

This study was an eight-week feasibility pilot study with a randomized controlled trial design. As all aspects of the trial (recruitment, assessment, treatment allocation and intervention) were executed by one single person (A.J.), no blinding for group allocation was performed. Data were collected at the Department of Physiology of the Anton De Kom University of Suriname. A questionnaire was developed to obtain information on age, sex, ethnicity, dominant side, affected body side, type of stroke, time post-stroke, presence of diabetes, hypertension, chronic obstructive pulmonary disorders and smoking status. These variables together with measures of exercise tolerance, physical activity (PA) and exercise self-efficacy were collected at baseline (T0), after 4 weeks (IG only) (T4) and after 8 weeks (T8).

### Feasibility

Evaluation for feasibility of the intervention was based on [29]: (1) how many participants finished the intervention (adherence); (2) preliminary effectiveness; (3) adverse events due to the intervention (safety) and (4) patient satisfaction and perception of the intervention.

Patient satisfaction and perception was evaluated using a self-developed questionnaire consisting of 7 questions evaluating how satisfied they were with the different parts of the program, to be rated on a 5-point scale: 1, very unsatisfied; 2, unsatisfied; 3, don’t know; 4, satisfied; 5, very satisfied. The questionnaire finished with an optional open question “What would you recommend us to make our program better?”.

### Clinical efficacy

Primary outcome measure of efficacy was functional exercise tolerance assessed by the 6MWT [30]. For this test, participants were instructed to walk continuously at their fastest pace for 6 minutes on a hallway of 30m with turning points at each end. They were told to cover as much ground as possible. Standardized verbal reassurance was given during the test. Subjects were allowed to use assistive devices (e.g. cane) and to rest when needed and chairs were placed at each end of the hallway and in the middle. All participants received the same standardized instructions and encouragement. The maximal distance covered in six minutes was recorded in meters.

Functional variables further included the Disabilities of the Arm, Shoulder and Hand (DASH) score, maximal handgrip (HG) strength, and the Berg Balance Scale (BBS). The DASH questionnaire [31] is a 30-item self-report questionnaire to assess disabilities in the upper extremity. Higher scores indicate more severity of symptoms and disability. Handgrip strength was measured using a hand dynamometer with an adjusted handle (Suahan Digital SH5003, Korea). The upper arms were supported next to the body, with the elbow joint in 90° degrees. The participant performed the maximal handgrip test 3 times alternating the paretic (P) and non-paretic (NP) arm with intervals of 30 seconds of rest [32]. The highest value for each hand was recorded. Balance control was evaluated by the BBS, consisting of 14 tasks and a score range of 0–56 [33].

The SCI exercise Self-Efficacy Scale (ESES, Dutch version), consisting of 10 items to be scored on a 4-point-likert scale, was used to obtain information on exercise self-efficacy [34].

### Interventions

Participants were randomized on a 2:1 ratio to either the intervention group (IG) or usual care (UC), using a computerized random number generator (www.randomization.com). The group allocation list was kept with the supervisor (R.B.) and every new inclusion was consequently added and allocated to the group as indicated in this list. Although initially a 1:1 randomization was planned, after discussing the resources for the testing following delays due to equipment issues, the number of patient tests was reduced by shifting to a 2:1 randomization. The 2:1 randomization allowed to gather as much information as possible on feasibility and safety of the intervention program while still collecting data for exploratory comparative analysis. Participants in the IG received a home-based, semi-supervised physiotherapy program. The CG received usual care. At this moment usual care for chronic stroke patients in Suriname consists of ten physiotherapy sessions (with only 54% of the costs per session are refundable) a year for people insured by the largest governmental health insurance company. So, by the time people after stroke are in the chronic phase after stroke, they do not have any therapy sessions left for that year. Moreover, if no physiotherapy is requested by the patient, none is given which is most often the case.

Overall, the intervention employed a holistic approach [35] bringing together physical fitness improving exercise, functional exercise, upper limb exercise as well as patient education according to the needs of the patients (Table 1). The home-based physiotherapy program included stair climbing, sit-to-stand exercise [4] and walking [36]. Upper extremity exercises were incorporated in the start-up and ending phases of each training session and consisted of proprioceptive neuromuscular facilitation techniques and mobility exercises. All treatment sessions between T0 and T4 (supervised phase, 3 times/week, 70 minutes/session) were supervised by a physiotherapist. During these first 4 weeks, exercise intensity was monitored using a Garmin Forerunner 225 watch on the non-paretic arm for heart rate measurements [37] along with a BORG scale [38]. The number of steps were measured using a pedometer (Yamax Digiwalker SW-200, Japan) [39] placed on the non-paretic leg at the level of the knee [40] and step counts per training session were noted. In addition, blood pressure (BP) measurements were done before, during and after the sessions in sitting position in order to monitor blood pressure response to exercise and ensure safety. At the end of the first 4 weeks, patients were reassessed for the above-mentioned efficacy outcome measures in order to track progress and adapt the exercise intensity accordingly. Between T4 and T8 (tele-coaching phase), the instructions were to continue the same individually tailored program without supervision, with help from the participants’ usual caregiver if necessary, and with a progressive exercise volume defined by duration and BORG scale as well as a pedometer step goal [41]. Patients were asked to log their performance in a diary, logging exercise durations, exercise intensity (BORG scale) and step counts. The physiotherapist provided weekly telephone encouragements and instructions.

**Table 1:** Overview intervention program.

Duration	Training phase**	Content of intervention	Progression of intervention
10–15 min	Starting up (putting on devices such as Yamax pedometer, Garmin watch; measuring resting blood pressure***).	Mobility exercise: Slow marching in one place, ankle circles; Shoulder, hip circles (slowly); Shoulder, elbow, wrist, hand and fingers movements slowly (all in the direction of PNF techniques)	Progression of upper limb exercises were based upon individual responses to the intensity.
40–45 min	Lower limb strengthening endurance exercise (As patients progressed, the number and length of rest periods were also decreased. As performance improved tasks were made more challenging (complexity of a task, number of repetitions, in various body positions).)	Stair climbing exercise: (intensity increased gradually after first 5 minutes of exercise with perceived exertion 4–5 out of 10 on BORG scale)	During stair climbing manual assistance was reduced as necessary for safe climbing without an orthosis
		Resting period (2-5min)	
		Sit-to-stand exercise: (2 sets of 5 and gradual increase to 3 sets of 10 with variable speed)	As the patients progressed resting periods during sit-to-stand exercise was decreased.
		Resting period (2-5min)	
		Walking (with or without assistive device): (amount of steps as target, with gradual increase in intensity, based on perceived exertion 4–5 out of 10 on BORG scale)	As the patient progressed, walking was done without an assistive device. Balance was challenged during walking by using ankle strategies. Walking was conducted on various surfaces including hardened ground and grass. During walking emphasis was on instructions including walking kinematics such as equal strides, cadence, weight shift, arm swing and equal limb loading.
		Resting period (2-5min)	
10–15 min	Ending program: upper limb rehabilitation, patient and family education^ (taking off devices; measuring resting blood pressure***)	Mobility exercise (including repositioning of shoulder where needed), PNF techniques for upper and lower limb followed by slow stretching. Information about stroke etiology and progression of stroke symptoms together with information on food choices and comorbidities such as high blood pressure and diabetes.	

*PNF, Proprioceptive Neuromuscular Techniques; min, minutes;

**Based on the Health Navigator Model & Chronic Care Model;

*** Standardized blood pressure measurements;

^Based on the Transtheoretical model.

### Data analysis

Database management was performed by use of Microsoft Excel 10.0. Intention to treat analysis was performed and therefore all patients who were enrolled and who were randomly allocated, are also included in the analysis, and are analyzed in the group to which they were randomized. Imputations for missing data among primary (6MWD) and secondary (DASH score, handgrip strength and Berg Balace Score) outcomes, were performed using mean substitution.

Given the small sample size and the increased risk for type 2 error inherent to ITT analysis, an additional per-protocol analysis was performed as well, allowing results from both analyses to be compared in order to strengthen our findings.

The Shapiro-Wilk test was used to determine normal distribution of the data. The groups were compared to evaluate differences in demographic, anthropometric and clinical parameters using unpaired t-tests or chi square test as appropriate. Exploratory evaluation of differences between and within CG and IG after 8 weeks was performed by means of 2-way mixed methods design analysis of variance with time as a repeated-measures factor. Effects sizes were calculated by means of partial eta square as obtained from ANOVA. SPSS version 21.0 was used to perform statistics. Statistical significance was set at p<0.05.

Patients or the public were not involved in the design, or conduct, or reporting, or dissemination plans of this study.

## Results

### Participants

**Fig 1:**
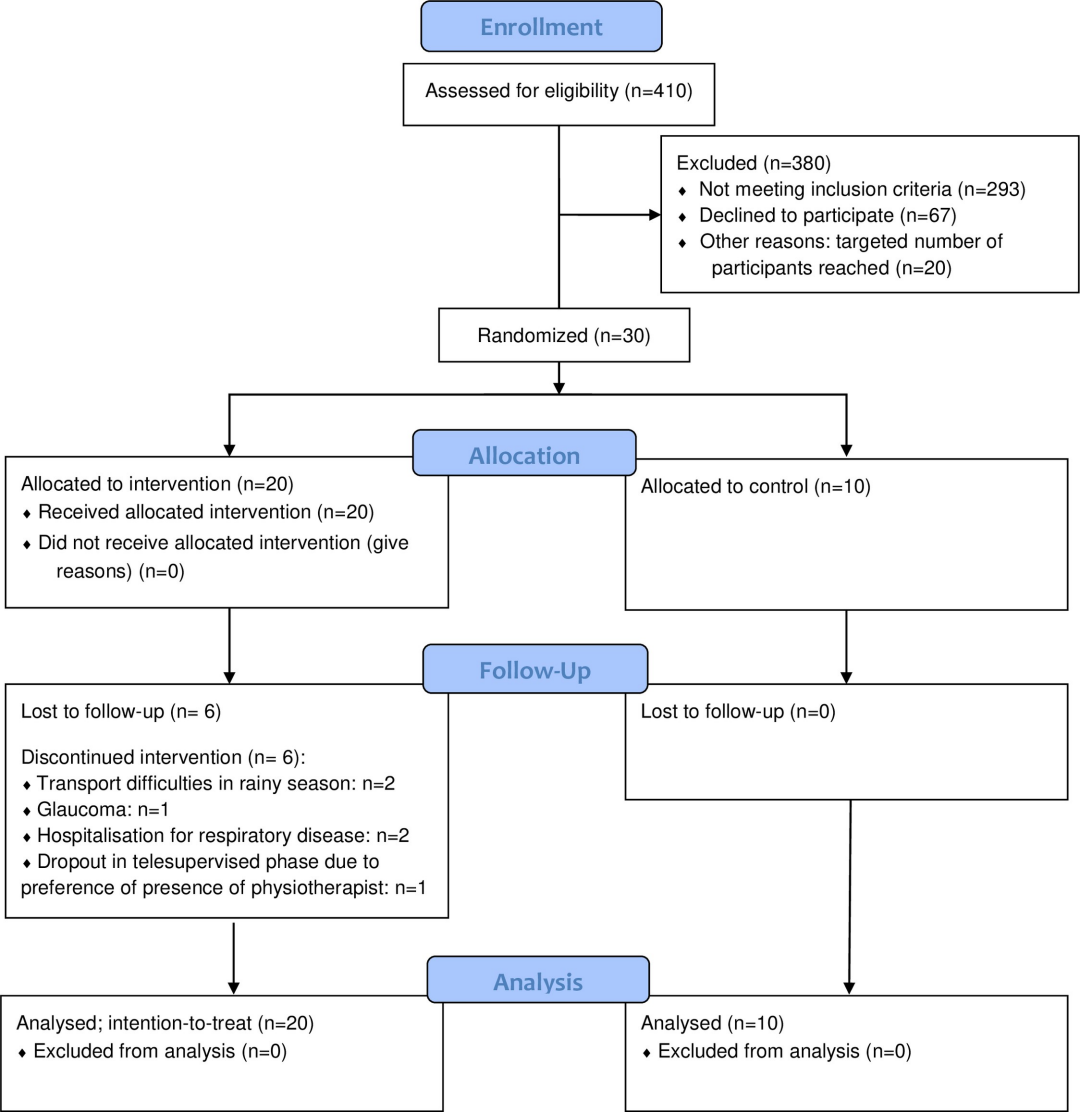
Flow of the SunRISe study.

The study flow is shown in Fig 1. Out of fifty-nine patients who were eligible for participation, the first thirty patients that agreed to participate, were randomized to either IG (n = 20) or CG (n = 10).

Demographic and clinical characteristics of the participants are shown in Table 2. The mean age of the participants was 61.8 ± 9.2 and 43% were men. Most participants had suffered ischemic stroke and were from Asian background. The mean time post-stroke was 3.3 years. At baseline, no significant differences were present between IG and CG, apart from the presence of diabetes, which was higher in the control group (p = 0.045). None of the participants were on a regular schedule of physiotherapy, therefore control group participants did not receive any exercise therapy in the 8-week timeframe of the study.

**Table 2:** Demographic and clinical characteristics of the study population.

	All	IG	CG
	N = 30	N = 20	N = 10
Sex (N, male)	13	9	4
Age (mean ± SD, years)	61.8 ± 9.2	61.6 ± 9.1	62.2 ± 9.1
Time post-stroke (mean ± SD, years)	3.3 ± 3.4	3.1 ± 3.5	4.5 ± 3.1
Type of stroke (N)			
*Ischemic*	27	18	9
*Hemorrhage*	3	2	1
Ethnic background (N)			
*Asian*	16	10	6
*African*	9	7	2
*Other*	5	3	2
Recurrent stroke (N, yes)	8	5	3
Affected body side (N, right)	17	13	4
Dominant side (N, right)	27	18	9
Diabetes (N, yes)	17	9	8
COPD (N, yes)	3	3	0
Hypertension (N, yes)	25	16	9
Former smoking (N, yes)	13	8	5
Current smoking (N, yes)	6	3	3
Current alcohol use (N, yes)	8	5	3

*CG, Control Group; COPD, Chronic Obstructive Pulmonary Disease; IG, Intervention Group.

### Feasibility

#### Adherence to the program

Fourteen of the twenty (70%) IG participants completed the full intervention (Fig 1). Drop outs during the supervised phase of the intervention were because of the rainy season (N = 2), sudden development of glaucoma (N = 1; diabetes patient) and hospitalization for respiratory problems (N = 2; history of COPD). One drop-out (N = 1) during the tele-supervised intervention was due to a preference for supervised physiotherapy. One participant reported to need help from a relative for attaching the pedometer for the tele-supervised sessions.

**Fig 2:**
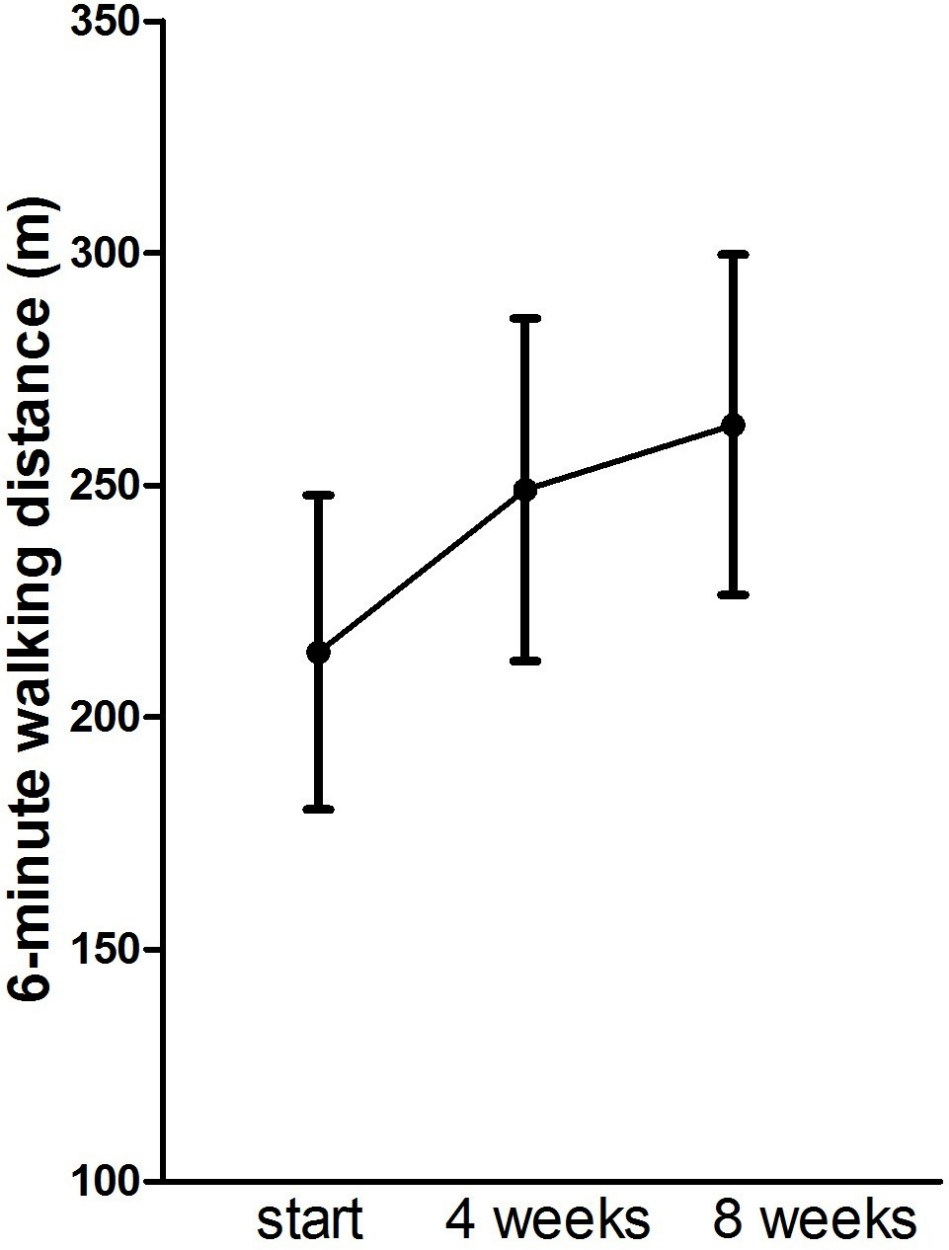
Changes in the primary outcome measure within the intervention group.

During the rehabilitation sessions, the mean number of steps gradually increased from 2026 ±1352 steps during the first session to 2990±1617 steps in the last week of the intervention as is also reflected by the 6MWT results half-way and at the end of the intervention (Fig 2).

#### Adverse events

No adverse events related to physical activity were noted during the study.

#### Patient satisfaction and perception

In Table 3, the results for patient satisfaction are provided. Participants generally experienced the program positively. A longer duration of the program/sessions would have been preferred by some of the participants.

**Table 3:** Patient satisfaction and perception.

Number	Question	Mean T8
1	How satisfied are you with the overall design of the exercise program?	4.7
2	How satisfied were you with the duration of the treatment sessions?	4.6
3	How satisfied was the composition of the treatment sessions?	4.7
4	How satisfied were you with the offered exercises?	4.7
5	How satisfied were you with the physiotherapist?	5
6	How satisfied were you with the pedometer?	4.7
7	How satisfied were you with the telephone support?	4.8
8**	What would you recommend us to make our program better?	1. *“the program is good and can be longer as these are lifelong exercises and since I’ve had this program my life has changed*.*”* (#2)
		2. *“the program can be longer because the exercise is very good for us” (#5)*
		3. *“Wish that the session was longer in order to give more attention to the arm exercise” (#1)*

*1, very unsatisfied; 2, unsatisfied; 3, don’t know; 4, satisfied; 5, very satisfied; T8, after 8 weeks of intervention.

**Question 8 includes patient perception and the number of patients with this comment between brackets.

#### Clinical efficacy

Baseline and follow-up measures are provided in Table 4. With regards to 6MWD, the IG started at a non-significantly lower level (227 ± 125m) compared to the CG (302 ± 117m), and increased their walking distance with 57.2 ± 67.3m which was significantly more than the CG (0.6±29.2m) (interaction effect; F(1, 28) = 6.36, p = 0.018, ŋ_*p*_^*2*^ *=* 0.185, power = 0.683).

**Table 4:** Changes in outcome measures.

	Intervention group (N = 14)		Control group (N = 10)		Group effect	Time effect	Group*time interaction effect
	Baseline	Week 8	Baseline	Week 8			
**Walking**							
6MWD (m)	227±126	285±143	303±117	303±129	0,310	0.192*	0.185*
**Upper extremity function**							
DASH score	41.0±23.8	31.2±19.8	33.9±26.6	34.5±25.8	0,002	0,113	0.140*
Handgrip paretic hand (kg)	11.7±8.9	13.8±10.0	19.5±15.8	19.4±15.3	0,074	0,038	0,048
Handgrip non paretic hand (kg)	29.6±8.3	29.7±9.3	29.5±8.8	27.9±10.6	0,003	0,019	0,025
**Balance**							
BBS score	50.8±4.5	54.3±4.4	51.4±7.0	53.0±6.4	0,001	0.308*	0,056

*Significant result at p < 0.05.

MWD, Six-Minute Walking Distance; DASH, Disabilities of the Arm, Shoulder and Hand; BBS, Berg Balance Score; ESES, Exercise Self-Efficacy Scale.

An interaction effect was present for the total DASH score (F(1, 28) = 4.54, p = 0.042, ŋ_*p*_^*2*^ *=* 0.140, power = 0.539) indicating a signicant greater improvement in the IG (33 ± 26) than in the CG (0.6±2.3). There was a trend towards improvement in the BBS, paretic and non-paretic HG, but compared to the CG no significant differences were found. Mean self-efficacy score was 34.45 during baseline measurements, no difference with the CG was present. After performing per protocol analysis for the same variables, results remained similar. For 6MWD and DASH, significant group*time interaction effects were found (effect size of 0.203 and 0.133 respectively; p<0.05). No other interaction effects were present. Significant time effects were found for 6MWD and BBS score (effect size of 0.210 and 0.308 respectively; p<0.05).

## Discussion

In this study we documented the feasibility of a self-developed 8-week home-based semi-supervised physiotherapy intervention to promote functional exercise tolerance, UE mobility and functional balance in chronic stroke survivors living in a LMIC setting.

### Feasibility

Fourteen of the twenty people enrolled in the program completed the full program which equals a 70% successful participation rate. Other feasibility studies [42, 43] with chronic stroke patients reported similar participation numbers after testing home-based or tele-physiotherapy programs. Our IG had a high exercise self-efficacy. Studies show that self-efficacy is important to stimulate and maintain PA behavior. Haworth et al. [44] demonstrated that self-efficacy might increase apart from an intervention program, which could be seen in our CG.

As could be expected, we had a few dropouts in the IG. The dropouts in the supervised phase were all the result of infrastructural problems and health issues, and not directly related to our intervention program. An environment where the walking area could better be used in rainy conditions and where the sewerage would protect against overflow of access water [45], would further have increased the feasibility of this intervention. It seems warranted that improved infrastructure and safe walking areas should be part of an integral stroke-care plan [45] in the LMIC, since a simple exercise such as walking might be enough to improve functional exercise tolerance post-stroke [46]. Additionally, more regular medical follow-up after hospital discharge, would improve overall health and increase participation in secondary prevention programs, which should also be an important focus in developing countries as prevention generally is a cost saving strategy in the long run.

A drop out in the tele-supervised phase may indicate a need for further individualization of the home-based program [47] and/or a preference or a need for more supervised therapy sessions before entering the tele-supervised phase.

### Adverse events

None of the participants experienced any adverse events, despite that more than half of the study population presented with diabetes and hypertension and/or used diuretics, B-blockers, and/or statins [48].

### Acceptability

As exercise was one-on-one in both phases, this intervention can be categorized as labor intensive, but the simplicity of the intervention (requiring no equipment, no expensive technology nor special education) made it possible for the caregiver to help with supervision, especially during the tele-supervised phase. The physiotherapy program was easily fitted in each participants’ house and neighborhood. Hence, the participants perceived the intervention as supportive. This follows the rationale that a persons’ functioning is the result of dynamic interactions between several factors, such as health conditions and environmental factors [49].

### Clinical efficacy

Results from both the intention-to-treat and the per protocol analysis were in line with each other, documenting significant effects in 6MWD and DASH score. The observed improvement in 6MWD in the IG (57m) was similar to findings from other home- and community-based studies (range 45-85m) [19, 50]. The minimal clinical important difference for the 6MWD in patient studies with stroke is between 34–50 m [51, 52] so it can be concluded that the program was effective in improving walking capacity. Our study had a power of 0.6 with an effect size of 0.18. In order to confirm our findings, a full scale RCT with a power of 0.8 would need twenty-three participants in each treatment group in order to be able to reject the null hypothesis (type I error probability 0.05).

Also consistent with the findings of Gordon et al. [46], the IG in our study increased in their distance walked, which then has implications for functional ambulation and participation in the community. We expected this improvement in the IG. However, we did not find any effect on paretic and non-paretic HG. This might be explained by UE functional recovery, which appears to be slower than that of the lower extremity [53]. Moreover, both groups show a large SD for the paretic and non-paretic HG strength, which might be a consequence of our small sample size. The latter might also explain why we did not find any statistical difference between IG and CG for this variable. Considering the effects found in the DASH score and HG strength (at baseline and after the program), the fact that HG strength represents overall physical strength [54, 55], and its influence on functional exercise tolerance [56], we advise a full scale RCT to include both of these measurements to further investigate this.

At last, the improvement of functional balance alongside functional exercise tolerance in the IG, can be explained by the correlation between these two measurements in post-stroke patients [57]. But, compared to the CG we did not find a difference for functional balance between groups, as they both seemed to be at the same level after 8 weeks. And, possibly, the BBS reached its ceiling effect in this study group making it difficult to evaluate balance improvement [58] between groups. A future trial should probably look into a different strategy to assess balance as this is an important aspect of physical fitness and important in the prevention of falls.

### Limitations and strengths

Ideally, a full-scale RCT should stratify for age and sex in order to exclude effects of these factors. In the context of Suriname, ethnic background might also need to be considered in the stratification as cultural differences might warrant therapeutically different approaches. In our study no baseline differences were found for these factors. In a powered efficacy trial, it is also advised to have a T4 measurement for the CG, but because of limited resources along with the main objective of evaluating feasibility of the full intervention, we decided to exclude this.

The study measurements were not performed by a blinded researcher as the full study was executed by one investigator, assisted by master students. Although all possible efforts were made to conduct the measurements in a standardized manner, potential bias from knowing the group allocation cannot be ruled out.

The 6MWT was an appropriate alternative to measure exercise tolerance compared to the golden standard, peak oxygen uptake measured by a symptom-limited graded cardiopulmonary exercise test (CPET). Initially, peak oxygen uptake was set as primary outcome of this study, however due to technical problems and unavailability of timely technical support and resources for repair of this lab infrastructure, this evaluation could not be performed in the majority of participants. Therefore, results from the 6MWT also included in the protocol, were used to evaluate the primary outcome measure of exercise tolerance. The 6MWT requires minimal equipment, was conducted in our institute by paramedical personnel and without any adverse events. The self-efficacy questionnaire in this study was originally constructed for the spinal cord injury population. Haworth et al. [44] used a self-constructed exercise self-efficacy scale (ESS) in a group with stroke and SCI patients (grouped as patients with acquired neurological pathologies). A part of the encountered stroke population during the recruitment phase was not fit enough for this intervention, hence an adapted program for them might be needed and our program and study results cannot be generalized to the entire chronic stroke population. Furthermore, as time of chronicity showed large variability and a maximum time of chronicity was not included in the eligibility criteria, there may have been large variability of health conditions between participants as well. However, all participants could benefit from physiotherapy. Similarly, our study results do not allow for drawing conclusions as to whether or not differences in comorbidities in the study groups have influenced intervention efficacy.

## Conclusions

Our findings suggest that our home-based semi-supervised physiotherapy intervention is safe, associated with moderate to high levels of engagement and patient satisfaction and induces walking improvement. However, conducting a full-scale RCT might imply the need to tackle infrastructural problems int his LMIC. Larger studies are warranted for confirmation of efficacy, cost-effectiveness and ethnic/cultural effects.

## Supporting information

S1 Protocol Protocol of the SunRISe-study as approved by the ethics committee. (PDF)

S1 Checklist (DOC)
