# Supplementary material for: Feasibility of a home-based physiotherapy intervention to promote post-stroke mobility: A randomized controlled pilot study
Source: PLoS One. 2022 Mar 7;17(3):e0256455. doi: 10.1371/journal.pone.0256455 (PMC8901054; doi:10.1371/journal.pone.0256455)
Supplement: S1 Protocol — (PDF) [file pone.0256455.s001.pdf]

The effect of a tele-supervised home based rehabilitation program on physical fitness, quality of gait, upper limb disability and quality of life in people after chronic stroke in an urban setting in Suriname

Protocol for a randomized controlled trial

Research team:

- A. Jarbandhan, M.Sc.; Faculty of Medical Sciences, Anton de Kom University of Suriname
- Dr. R. Buys; Department of Cardiovascular Sciences and department of Rehabilitation Sciences, KU Leuven, Belgium
- Prof.dr. H.E.J. Veeger; Department Biomechanical Engineering, Delft University of Technology, Netherlands
- Prof.dr. L. Vanhees; Department of Rehabilitation Sciences, KU Leuven, Belgium

Location:

Anton de Kom University of Suriname
Faculty of Medical Sciences
Physical Therapy Program
Paramaribo-Suriname

Introduction

In Suriname, Cardiovascular Disease (CVD) is the highest cause of death [1]. Stroke, which is part of CVD, is a major cause of adult disability and the number of stroke survivors in low and middle income countries such as Suriname (LMICs) is projected to rise [2]. Impaired upper (e.g. shoulder mobility) and lower extremity function (e.g. impaired ankle mobility) is common after stroke. Problems with motor control, sensory disturbances (including pain), problems using or understanding language (aphasia), depression, cognitive problems, changes in perception and fatigue syndrome can occur after stroke. These impairments result in decreased aerobic capacity, decreased walking capacity, decreased physical functioning and a deterioration of the quality of life.

It is well known that aerobic capacity is decreased in chronic stroke survivors [3] and that the level of physical activity in this group of patients is not enough to induce health benefits [4], which in turn increase their risk of early mortality and recurrent stroke [5]. Studies show that physical fitness training is a way to modify and improve aerobic capacity after chronic stroke [5, 6]. It is reported that an improvement in physical fitness is associated to an increase in sensorimotor function, improved walking capacity and increased quality of life in chronic stroke survivors [5-7][24]. However, so far, most studies were conducted in a laboratory or hospital setting. For chronic stroke survivors, home-based rehabilitation is a promising alternative to regular center-based stroke rehabilitation [8] especially after hospital- and community-based studies consistently reveal low levels of physical activity for stroke survivors [9, 10]. Literature search documents that the long term training effect and the effective training dose for aerobic capacity after home-based training for chronic stroke survivors is understudied [11]. A study done by Duncan and colleagues in a home-based setting reports significant gains for peak aerobic capacity after progressive home based exercise for patients with subacute stroke [12]. Moore and colleagues studied chronic stroke patients and showed significant increase in physical function, cognition and quality of life after a community-based exercise program[13]. Little is known regarding potentially beneficial effects of supervised home-based exercise training in patients with chronic stroke, warranting further research. [11, 13]

Similarly, impaired upper extremity function (e.g. reduced upper arm function and shoulder pain) is common after stroke and can be positively influenced by rehabilitation exercises [14]. In most high income countries, patients with chronic stroke keep visiting their physiotherapists for upper limb rehabilitation in order to maintain as much as possible the function and flexibility in the paralyzed arm, while also preventing or treating shoulder pain. In Suriname, patients with chronic stroke do not receive any further physiotherapy treatment after the acute phase of stroke. The enhancement of the functionality of the upper limb is a core element of stroke rehabilitation to maximize the effect on patient outcomes and decrease disability and is still considered effective in the chronic phase [15, 16]. Unfortunately, the financial resources are not in place in Suriname for patients in the chronic patients with stroke, making it unable for them to pay for further necessary treatments.

Telerehabilitation is a good way to reduce costs in a setting where the financial resources are low. It is defined as the ability to provide distance support, evaluation and intervention to persons who are disabled via telecommunication [17]. Literature reports [18, 19] some positive effects of telephone interviews on the motor function and quality of life in chronic stroke patients, but the effect on physical fitness level and adherence to a physical fitness program is understudied.

All the above summarized scientific evidence results from research in high income economies and the need for research in the setting of LMICs has already been outlined [20]. Reasons for the lack of trials in LMICs probably include funding difficulties and paucity in skilled personnel for research, but local conditions and characteristics of patients with stroke in these areas are equally important [21].

We can assume that patients in LMICs will experience more barriers towards participation in clinical exercise programs. Therefore the study designs adopted in previous research might not entirely suit the multi-ethnic

population of the LMIC of Suriname. Bringing an intervention as close as possible to the patient, while keeping the expenses low with help from emerging and relatively cheap technologies, seems more important in the setting of Suriname in order to avoid patients dropping out. We believe a home based physical fitness and rehabilitation program might be an effective way to stimulate chronic stroke survivors in Suriname to build long lasting healthy exercise habits resulting in long term positive effects on aerobic capacity, health related quality of life, quality of gait, and upper limb functionality.

Objectives

The main objective of this research is to investigate the short and longer term effects of 8 weeks of a tele-supervised home-based exercise and physiotherapy program on **aerobic capacity**, **quality of gait** and **health related quality of life** in people with chronic ischemic stroke.

At first, the feasibility and cost-effectiveness of the program will be evaluated.

Secondly, possible underlying determinants for aerobic capacity and the training effect on aerobic capacity will be investigated.

Methods

Study design: The proposed project is a prospective, single blind, randomized controlled intervention trial in patients with chronic stroke.

Setting: Patients will be recruited from the Rehabilitation Center Paramaribo; from the community and from the union for people after stroke.

Patient population: Patients with chronic ischemic stroke and following the inclusion criteria can be included in the study:

Inclusion criteria:
- ability to give informed consent
- ability to understand measurement procedures defined as MMSE >24 [22]
- last stroke >6 months ago

- mild to moderate stroke deficit defined by Fugl Meyer test score of 27 to 90 for upper and lower extremities [12]
- FAC ≥3
- medical clearance to participate in a moderate exercise program
- living at home and having a minimum of 5 stairs at home

Exclusion criteria:
- serious cardiac condition [22]
- other serious end organ damage
- other neurological deficits leading to disability
- uncontrolled blood pressure (systolic pressure >140, diastolic pressure >90) [22]

Statistical power and sample size: Sample size calculation was performed with PS Power and Sample Size Calculation (http://biostat.mc.vanderbilt.edu/PowerSampleSize) The sample size for the two-sample case (training versus control) was estimated on the basis of the results of Moore et al [13] for effect of exercise on cardiorespiratory capacity. In this study the response for peak VO_2 within each subject group was normally distributed with standard deviation 3.4. If the true difference in the experimental and control means is 3.0, we will need to study 17 experimental subjects and 17 control subjects to be able to reject the null hypothesis that the

population means of the experimental and control groups are equal with probability (power) 0.8. The Type I error probability associated with this test of this null hypothesis is 0.05. We chose to aim for a sample size of 20 patients per group taking drop outs into account.

Participant stratification will be balanced for gender because men tend to have higher aerobic capacity and muscle strength than women. The participants will be assigned randomly to a group by a computer program (www.randomization.com). Informed consent (appendix I) must be signed by every participant.

Intervention There is an intervention group (physical fitness exercise) and a control group (usual care) [13].

The intervention employs a holistic approach bringing together physical fitness improving exercises and upper limb exercises. The intervention is adapted from earlier studies in which physical fitness exercise showed a positive effect on cardiorespiratory capacity [12, 13] in chronic stroke survivors, along with earlier research experience on upper limb rehabilitation for stroke survivors [19]

The core of the exercise program will consist of three main exercises: stair climbing, chair rises [12] (is considered a balance exercise also) and walking [13].

At this moment Suriname does not have a structural exercise program in place for chronic stroke survivors. For this reason the usual care will exist of no exercise.

Table 1. Overview Intervention Group activities

Intervention group		
15 min	Warming up	Hip circles<br>Knee circles<br>Ankle circles<br>Slow marching<br>Stretching (gluteus, quadriceps femoris, hamstrings, triceps surae)<br>Shoulder circles<br>Shoulder abduction/adduction<br>Elbow extension/flexion<br>Wrist extension/flexion
40 min	Functional strengthening lower limbs [increase intensity gradually after first 5 minutes of exercise with perceived exertion 4-5 out of 10 on BORG scale]<br><br>[start with 2 sets of 5 and gradually increase to 3 sets of 10 with variable speed]<br><br>[use amount of steps as target and gradually increase to more to complete in certain time limit in which perceived exertion should be 4-5 out of 10 on BORG scale]	Stair climbing<br><br>Sit-to-stand<br><br>Walking
15 min	Cooling down (combination stretch exercise upper and lower limb)	Mobility exercise for upper and lower limb followed by slow stretching (upper extremity and the same muscle groups of lower limb)

The intervention will provided be by a physical therapist for 4 weeks (3 times/wk, 70 minutes). This duration of intervention was selected because it improved the cardiorespiratory function of chronic stroke survivors [11]. After that a telephone supervised method for 4 weeks is done to monitor if the patient is continuing the exercise program at a given intensity. To progress in cardiorespiratory fitness level a pedometer based step goal [4] will be set.

Measurements: Following measurements and paper and pencil surveys will be undertaken in order to evaluate the subjects at baseline at 4 weeks, 8 weeks and 5 months:

Anthropometric characteristics weight, height, body mass index, % fat (bio-electrical impedance) and waist and hip circumference will be measured.

Exercise tolerance (peak VO2) will be assessed by submaximal graded cardiopulmonary exercise testing (CPET) with breath-by-breath analysis (Jaeger Oxycon Mobile®) on a cycle ergometer. Submaximal fitness parameters will include 1st and 2nd ventilatory thresholds, oxygen uptake efficiency slope and the VE/VCO2-slope. We will use a graded protocol in which the patient starts with a 10 min warming-up session at zero workload [12] followed by workload increments of 10W every minute until maximal effort is reached or symptoms appear [3, 12]. Submaximal effort is defined as the inability to continue cycling at a pace of 50-60 revolutions per minute due to shortness of breath and/or leg fatigue.

Hemodynamic and vascular function at rest will be non-invasively assessed by means of the Nexfin device which will provide resting systolic and diastolic blood pressure, cardiac output, stroke volume and systemic vascular resistance. Furthermore brachial ankle index will be determined by blood pressure measurements at non-paretic upper arm and ankle. Finally arterial stiffness will be evaluated by use of the Arteriograph.

Lung function will be evaluated by vital capacity and forced expiratory volume in 1 second measured before starting the CPET, using the same equipment for breath by breath analysis.

Habitual physical activity will be objectively assessed during 7 consecutive days and nights by means of the physical activity device that will also be used to guide the intervention, designed to be worn constantly around the upper arm (like a watch) [23].

Walking endurance will be tested with the 6-minute walk test. This test is a reliable submaximal test of cardiorespiratory capacity in people with stroke [24, 25].

Quality of gait will be defined by spatiotemporal parameters such as step length, stride length, step width, paretic stance time and gait speed. These parameters will be recorded in a human movement laboratory by two camera's (Panasonic HDC-TM900 3 MOS HD camcorder and Canon powershot SX40 HS) which capture data that will be analyzed with Kinovea, an open source video tool.

The manual muscle testing (hand-held dynamometer), and handgrip strength test will be used to evaluate upper limb function [26-29] and knee extensor strength [30, 31].

Balance will be evaluated by the Berg balance scale, which consists of 14 tasks and a score range of 0-56 [32].

Health related quality of life will be evaluated using the SF-12 questionnaire [33-35].

Disabilities of arm, shoulder and hand will be evaluated by means of the DASH questionnaire [36, 37].

Exercise self-efficacy will be evaluated by use of the Dutch translation of the Exercise Self-Efficacy Scale (ESES) [38].

Database management will be performed by use of Microsoft Excel 10.0. All data will be anonymized before being processed. Repeated measures ANOVA will be performed by use of SPSS statistical software in order to reveal differences between control and intervention groups regarding the primary and secondary outcome measures.

Time Planning:
Start research project: March 1st 2016
Anticipated end of research project: March 31th 2017

There will be no charges for the patient and there will also be no extra costs for the rehabilitation center as all measurements will be performed by staff and equipment belonging to the research group.

References:

1. Punwasi, W., *Doodsoorzaken in Suriname 2010-2011*, Epidemiology, Editor. 2012, Bureau of Public Health: Suriname. p. 11-17.
2. Strong, K., C. Mathers, and R. Bonita, *Preventing stroke: saving lives around the world.* Lancet Neurol, 2007. **6**(2): p. 182-7.
3. Baert, I., et al., *Evolution of cardiorespiratory fitness after stroke: a 1-year follow-up study. Influence of prestroke patients' characteristics and stroke-related factors.* Arch Phys Med Rehabil, 2012. **93**(4): p. 669-76.
4. Baert, I., et al., *Are patients 1 year post-stroke active enough to improve their physical health?* Disabil Rehabil, 2012. **34**(7): p. 574-80.
5. Billinger, S.A., et al., *Physical activity and exercise recommendations for stroke survivors: a statement for healthcare professionals from the American Heart Association/American Stroke Association.* Stroke, 2014. **45**(8): p. 2532-53.
6. Potempa, K., et al., *Physiological outcomes of aerobic exercise training in hemiparetic stroke patients.* Stroke, 1995. **26**(1): p. 101-5.
7. Saunders, D.H., et al., *Physical fitness training for stroke patients.* Stroke, 2004. **35**(9): p. 2235.
8. Siemonsma, P., et al., *Determinants influencing the implementation of home-based stroke rehabilitation: a systematic review.* Disabil Rehabil, 2014. **36**(24): p. 2019-30.
9. West, T. and J. Bernhardt, *Physical activity in hospitalised stroke patients.* Stroke Res Treat, 2012. **2012**: p. 813765.
10. Gebruers, N., et al., *Monitoring of physical activity after stroke: a systematic review of accelerometry-based measures.* Arch Phys Med Rehabil, 2010. **91**(2): p. 288-97.
11. Marsden, D.L., et al., *Characteristics of exercise training interventions to improve cardiorespiratory fitness after stroke: a systematic review with meta-analysis.* Neurorehabil Neural Repair, 2013. **27**(9): p. 775-88.
12. Duncan, P., et al., *Randomized clinical trial of therapeutic exercise in subacute stroke.* Stroke, 2003. **34**(9): p. 2173-80.
13. Moore, S.A., et al., *Effects of Community Exercise Therapy on Metabolic, Brain, Physical, and Cognitive Function Following Stroke: A Randomized Controlled Pilot Trial.* Neurorehabil Neural Repair, 2014.
14. Chae, J., et al., *Poststroke shoulder pain: its relationship to motor impairment, activity limitation, and quality of life.* Arch Phys Med Rehabil, 2007. **88**(3): p. 298-301.
15. Kraft, G.H., S.S. Fitts, and M.C. Hammond, *Techniques to improve function of the arm and hand in chronic hemiplegia.* Arch Phys Med Rehabil, 1992. **73**(3): p. 220-7.
16. Hummelsheim, H. and C. Eickhof, *Repetitive sensorimotor training for arm and hand in a patient with locked-in syndrome.* Scand J Rehabil Med, 1999. **31**(4): p. 250-6.
17. Lai, J.C., et al., *Telerehabilitation - a new model for community-based stroke rehabilitation.* J Telemed Telecare, 2004. **10**(4): p. 199-205.
18. Johansson, T. and C. Wild, *Telerehabilitation in stroke care--a systematic review.* J Telemed Telecare, 2011. **17**(1): p. 1-6.

19. Laver, K.E., et al., *Telerehabilitation services for stroke.* Cochrane Database Syst Rev, 2013. **12**: p. CD010255.
20. Turk-Adawi, K.I. and S.L. Grace, *Narrative review comparing the benefits of and participation in cardiac rehabilitation in high-, middle- and low-income countries.* Heart Lung Circ, 2015. **24**(5): p. 510-20.
21. Anjos, S.M., et al., *Translational neurorehabilitation research in the third world: what barriers to trial participation can teach us.* Stroke, 2014. **45**(5): p. 1495-7.
22. Pang, M.Y., et al., *A community-based fitness and mobility exercise program for older adults with chronic stroke: a randomized, controlled trial.* J Am Geriatr Soc, 2005. **53**(10): p. 1667-74.
23. Liang, W., et al., *Habitual physical activity reduces the risk of ischemic stroke: a case-control study in southern china.* Cerebrovasc Dis, 2009. **28**(5): p. 454-9.
24. Eng, J.J., et al., *Functional walk tests in individuals with stroke: relation to perceived exertion and myocardial exertion.* Stroke, 2002. **33**(3): p. 756-61.
25. Eng, J.J., A.S. Dawson, and K.S. Chu, *Submaximal exercise in persons with stroke: test-retest reliability and concurrent validity with maximal oxygen consumption.* Arch Phys Med Rehabil, 2004. **85**(1): p. 113-8.
26. Sunderland, A., et al., *Arm function after stroke. An evaluation of grip strength as a measure of recovery and a prognostic indicator.* J Neurol Neurosurg Psychiatry, 1989. **52**(11): p. 1267-72.
27. Bohannon, R.W., *Dynamometer measurements of hand-grip strength predict multiple outcomes.* Percept Mot Skills, 2001. **93**(2): p. 323-8.
28. Ada, L., S. Dorsch, and C.G. Canning, *Strengthening interventions increase strength and improve activity after stroke: a systematic review.* Aust J Physiother, 2006. **52**(4): p. 241-8.
29. Ada, L., N. O'Dwyer, and E. O'Neill, *Relation between spasticity, weakness and contracture of the elbow flexors and upper limb activity after stroke: an observational study.* Disabil Rehabil, 2006. **28**(13-14): p. 891-7.
30. Bohannon, R.W., *Test-retest reliability of hand-held dynamometry during a single session of strength assessment.* Phys Ther, 1986. **66**(2): p. 206-9.
31. Bohannon, R.W. and A.W. Andrews, *Interrater reliability of hand-held dynamometry.* Phys Ther, 1987. **67**(6): p. 931-3.
32. Makizako, H., et al., *Use of the Berg Balance Scale to predict independent gait after stroke: a study of an inpatient population in Japan.* PM R, 2015. **7**(4): p. 392-9.
33. Suenkeler, I.H., et al., *Timecourse of health-related quality of life as determined 3, 6 and 12 months after stroke. Relationship to neurological deficit, disability and depression.* J Neurol, 2002. **249**(9): p. 1160-7.
34. Lim, L.L. and J.D. Fisher, *Use of the 12-item short-form (SF-12) Health Survey in an Australian heart and stroke population.* Qual Life Res, 1999. **8**(1-2): p. 1-8.
35. Muller-Nordhorn, J., S. Roll, and S.N. Willich, *Comparison of the short form (SF)-12 health status instrument with the SF-36 in patients with coronary heart disease.* Heart, 2004. **90**(5): p. 523-7.
36. Khan, W.S., et al., *The 'M2 DASH'-Manchester-modified Disabilities of Arm Shoulder and Hand score.* Hand (N Y), 2008. **3**(3): p. 240-4.
37. Baker, K., et al., *Measuring arm function early after stroke: is the DASH good enough?* J Neurol Neurosurg Psychiatry, 2015.
38. Nooijen, C.F., et al., *Exercise self-efficacy in persons with spinal cord injury: psychometric properties of the Dutch translation of the Exercise Self-Efficacy Scale.* J Rehabil Med, 2013. **45**(4): p. 347-50.
